# Neurocutaneous syndromes in art and antiquities

**DOI:** 10.1002/ajmg.c.31917

**Published:** 2021-05-20

**Authors:** Martino Ruggieri, Amalia Egle Gentile, Vincenza Ferrara, Massimo Papi, Andrea D. Praticò, Albert Mudry, Domenica Taruscio, Giuseppe Micali, Agata Polizzi

**Affiliations:** ^1^ Unit of Rare Diseases of the Nervous System in Childhood, Department of Clinical and Experimental Medicine, Section of Pediatrics and Child Neuropsychiatry University of Catania Catania Italy; ^2^ National Centre for Rare Diseases Istituto Superiore di Sanità Rome Italy; ^3^ Laboratories of Art and Medical Humanities, Faculty of Pharmacy and Medicine Sapienza University of Rome Rome Italy; ^4^ National Institutes for Health Migration and Poverty (NIHMP) and DermArt Rome Italy; ^5^ Department of Otolaryngology, Head and Neck Surgery Stanford University School of Medicine Stanford California USA; ^6^ Unit of Clinical Dermatology, Department of General Surgery and Medical and Surgical Specialties, Section of Dermatology and Venereology University of Catania Catania Italy; ^7^ Department of Educational Sciences, Chair of Pediatrics University of Catania Catania Italy

**Keywords:** artwork, disorders, neurocutaneous, painting, phacomatosis, print

## Abstract

Neurocutaneous syndromes are a group of genetic disorders affecting the skin, the central and peripheral nervous system, and the eye with congenital abnormalities and/or tumors. Manifestations may also involve the heart, vessels, lungs, kidneys, endocrine glands and bones. When people with these disorders are portrayed in works of art, physicians have speculated on possible diagnoses. In particular, many figures have been labeled as possibly having a neurocutaneous disorder, sometimes distorting the popular conception of these diseases. We review numerous documents, drawings, prints, lithographs, xylographs, and portraits which span the ages from antiquity to the era of the pioneers behind the eponyms, depicting a large spectrum of neurocutaneous disorders.

## 1 INTRODUCTION

### 1.1 Definition of the neurocutaneous syndromes

In their current definition, neurocutaneous syndromes/disorders (or phacomatoses) are a heterogeneous group of genetic diseases affecting the skin, the eye, and the central and peripheral nervous systems. Manifestations may involve many other organs and systems including heart, vessels, lungs, kidneys, endocrine glands, and bones (Gomez, 1987; Islam & Roach, 2015; Ruggieri, Pascual‐Castroviejo, & di Rocco, 2008). Nowadays, the most commonly used terms remain neurocutaneous syndromes and phacomatoses, which are used interchangeably.

### 1.2 Origin of the name(s)

**FIGURE 1:**
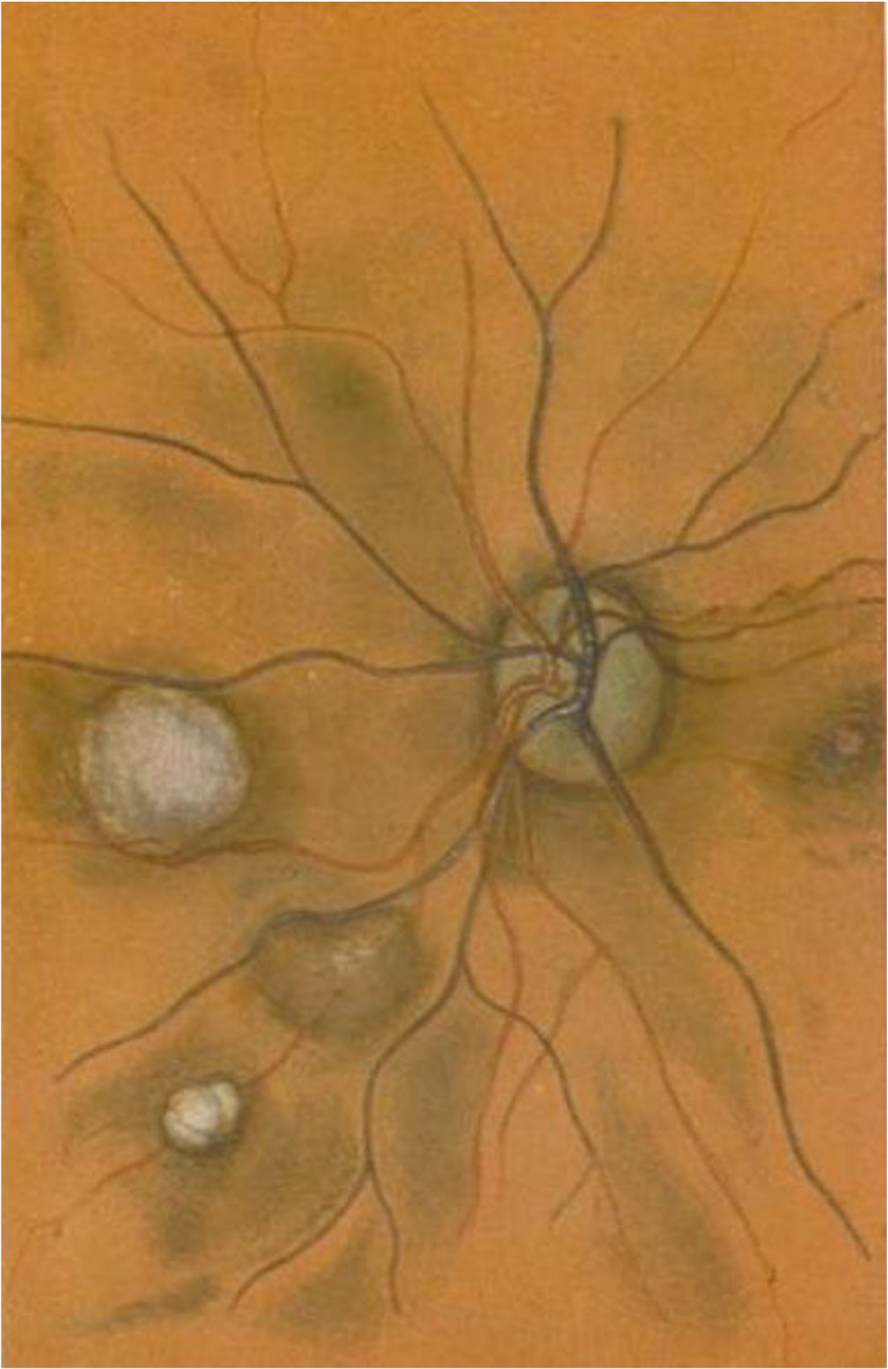
The original color print used by Jan van der Hoeve to depict the retinal “phakomas” seen as whitish/gray oval lesions in the central and left retina in the fundus of a patient with tuberous sclerosis. Copyright © The British Medical Council Library, London, UK

Three of these conditions, that is, von Recklinghausen neurofibromatosis (Von Recklinghausen, 1882), Bourneville tuberous sclerosis (Bourneville, 1880; Bourneville & Brissaud, 1881), and encephalotrigeminal angiomatosis of Sturge (1871) and Weber (1929), were grouped under the umbrella name of “phakomatoses” by the Dutch ophthalmologist Jan van der Hoeve [1878–1952] (Van der Hoeve, 1932, 1933), who had coined the term from the Greek word phakoma/phakomata, meaning lentil, spot, lens‐shaped, to define the retinal lesions recorded in tuberous sclerosis (Van der Hoeve, 1920, 1921) (Figure 1) and in neurofibromatosis (Van der Hoeve, 1923).

The synonym “neurocutaneous syndromes” came from the studies of the American neurologist Paul Ivan Yakovlev and the American psychiatrist Riley H. Guthrie. They established the key phenotypic and diagnostic roles of nervous system and skin in the complex medical conditions affecting some individuals seen at the Monson State Hospital for Epileptics in Massachusetts (Ashwal, 1990; Yakovlev & Guthrie, 1931). They named these disorders the neurocutaneous syndromes, or ectodermatoses, to explain the pathogenesis due to a common embryological origin from the neuroectodermal layer. The Belgian pathologist Ludo van Bogaert came to similar conclusions (Van Bogaert, 1935), but called them neuroectodermal dysplasias (Ashwal, 1990).

The Dutch pathologist Sylvan E. Moolten (Moolten, 1942) named Bourneville tuberous sclerosis “tuberous sclerosis complex”, thus recognizing its multi‐organ nature. He first introduced the terms hamartial, hamartoma, and hamartoblastoma, derived from the Greek verb hamartánein, which means “to miss the mark” or “to err” or “to fail.” Later, the American pediatric neurologist Manuel R. Gomez changed definitively the former concept of “phakoma/phakomata” into the more modern definition of “hamartia/hamartoma” (Gomez, 1987). Genodermatoses and neurocristopathies have been and still are sometimes used as alternative terms in the scientific literature (Ruggieri et al., 2020).

## 2 NEUROCUTANEOUS SYNDROMES IN WORKS OF ART

As works of art, neurocutaneous syndromes have stimulated a voluminous literature (Ashrafian, 2017; Lidden, 2006; Om & Om, 2018; Ponti et al., 2016; Ruggieri, Praticò, Caltabiano, & Polizzi, 2018; Ruggieri, Praticò, & Polizzi, 2020), which reflect wide differences of opinion (Ruggieri, Praticò, Scuderi, et al., 2017; Ruggieri, Praticò, Serra, et al., 2017; Ruggieri, Praticò, Catanzaro, Palmucci, & Polizzi, 2018). Only in the 19th and 20th centuries have these syndromes become better understood (Ashwal, 1990; Beighton & Beighton, 1986, 1997; Gomez, 1987; Ruggieri, Micali, & Praticò, 2018), even though the popular representations of affected individuals, both in real life (Cohen Jr., 1986; Howell & Ford, 1983) and in fiction (Cox, 1985; Solomon, 1968), created misconceptions and often conflated the various syndromes (Ruggieri, Praticò, Caltabiano, & Polizzi, 2018; Ruggieri, Praticò, & Polizzi, 2020).

This article will focus on selected artistic renditions from the old ages until contemporary times. We will list the artworks described herein according to the most recent classification of neurocutaneous syndromes (Ahn, Jackler, & Lustig, 1996; Huson, 1994; Ruggieri, Praticò, Caltabiano, & Polizzi, 2018; Ruggieri, Polizzi, et al., 2020a).

## 3 THE NEUROFIBROMATOSES

Three forms have been recognized under the umbrella term of neurofibromatosis, including neurofibromatosis Type 1 (NF1) (Von Recklinghausen, 1882), neurofibromatosis Type 2 (NF2) (Gardner & Turner, 1940; Wishart, 1822) and schwannomatosis (MacCollin, Woodfin, Kronn, & Short, 1996). These are autosomal dominant conditions caused by different genes, which share common clinical features, including hyperpigmented birthmarks of the skin, congenital anomalies (e.g., bone and/or vascular dysplasias), and a predisposition to develop benign and, less frequently, malignant tumors of the central and peripheral nervous system but also of other organs and systems (Ferner et al., 2019; Halliday, Parry, & Evans, 2019; Plotkin & Wick, 2018, Ruggieri et al., 2016).

### 3.1 Neurofibromatosis Type 1

#### 3.1.1 Multiple skin nodules in an enigmatic Hellenistic statuette [331–23 BC]

**FIGURE 2:**
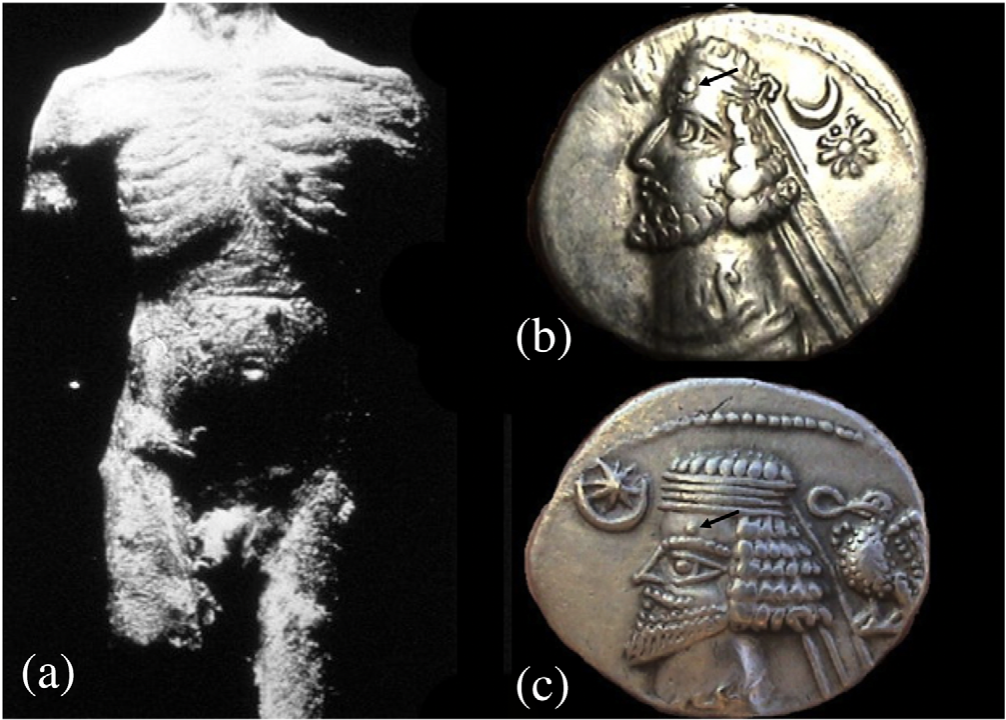
(a) Hellenistic statuette, representing a partial adult male body covered with multiple, smooth and sessile nodules, interpreted as neurofibromas. Copyright © 1994 Nature Publishing Group; (b,c) coins of the Parthian empire reproducing the left face of king Orodes II (b) [50 BC], and king Phraates IV (c) [20 BC] each represented with a large and round nodule, supposedly a neurofibroma, in their forehead (black arrows). Copyright © Archives of www.parthia.com (photographs by Douglas Mudd and Bart Lewis)

This statuette (Figure 2a) probably belongs to the Hellenistic period (323–31 BC) and originates from the Aegean town of Smyrna, in Anatolia. A healthy male with trunk and part of limbs is partially covered with multiple smooth, sessile nodules, resembling cutaneous neurofibromas. Some authors have suggested that it could have been a medical teaching aid while others hypothesized that it was more probably a decorative figure, maybe a satyr or a humanoid being, possibly a votive offer to the divinity (Gourevitch & Grmek, 1994; Linden, 1994; Ragge & Munier, 1994).

#### 3.1.2 Facial rounded nodules in the coins' effigies of the kings of Parthia [ca. 100 BC]

Parthia was located between northeastern Iran and Turkmenistan. Much of the knowledge on the aspects of the kings of Parthia arises from the coins (Daryaee, 2017; Faghfoury, 2020). Centuries before, Alexander the Great had established the typical pattern of the empire's coin: a right‐facing profile of a divinity on the obverse and a full‐length deity on the reverse (Faghfoury, 2020). This pattern changed after the third century BC, when the rulers' own portraits started to appear in their left facing profile (Selwood, 1971). The first Parthian king to start issuing coins was Mithridates I [171–138 BC] (Selwood, 1971; Todman, 2008). The kings' representations in the coins are characteristic and realistic and it is possible to identify changes in the hairstyles and beards (Faghfoury, 2020; Selwood, 1971; Widengren, 1983). In the coins representing Mithridates II [124–91 BC; coins issued in 100 BC circa], Orodes II [57–37 BC], Phraates IV [37–2 BC], and in many of the successive kings up to Vardanes II [58 AD], a large round nodule is clearly visible in the forehead (Figure 2b,c) (Ashrafian, 2011; Selwood, 1971; Todman, 2008). The presence of these nodules in many generations suggests a possible hereditary trait, like neurofibromas in NF1 (Ashrafian, 2011; Selwood, 1971; Todman, 2008). Other hypotheses include common warts, trichoepitelioma (Hart, 1966), or basal cell carcinomas in the case of Mithridates II (Ashrafian, 2011).

#### 3.1.3 A large mass in von Megenberg's “Buch der Natur” [1309–1374]

Konrad von Magenberg [1309–1374] was a Bavarian scholar and philosopher who wrote more than 30 treatises on theological, astronomical, physical, historical, and political issues. His *Buch der Natur*, the first book of natural history in the German language, was extremely popular and was printed seven times, in several editions, in the 15th century alone (Baltrusaitis, 1960; Lattis, 2007; Pfeiffer, 2012).

**FIGURE 3:**
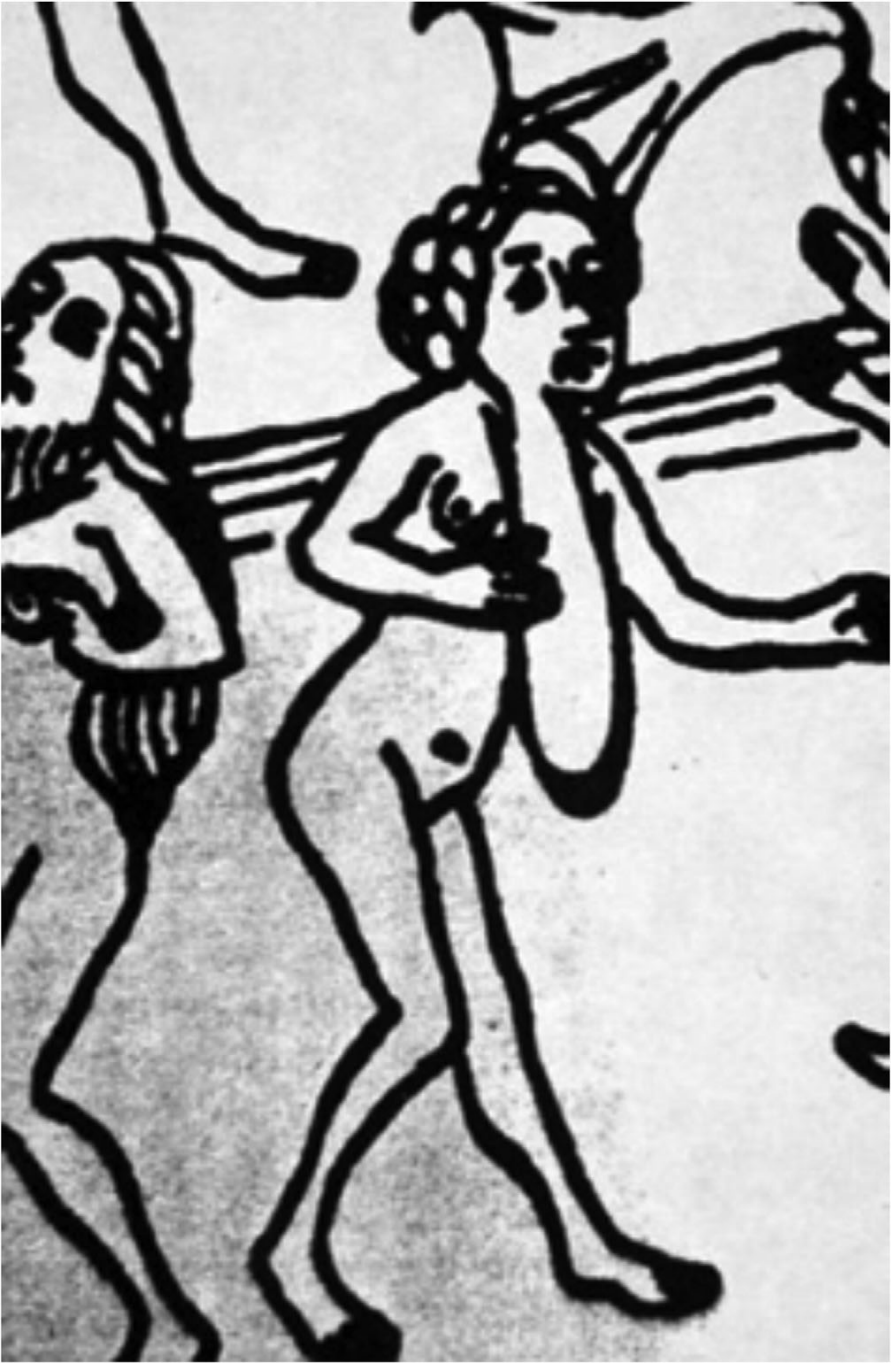
Close view‐up of a xylograph contained in the 12th Tome of Konrad von Magenberg's “Buch der Natur” [1475] representing a woman with large goiter, presumably a plexiform neurofibroma. Copyright © The Library of Congress, Washington, DC

In the 12th Tome of the Ausgburg edition [1475 AD], in addition to various types of imaginative figures including the mythological single large‐footed sciapod, the headless monster with six arms, the cynocephalus and the Cyclops, one can observe a woman (Figure 3) who, according to Choulant (1963), has a large goiter. According to Zanca and Zanca (1977, 1980) she has NF1 and to Ruggieri and Polizzi (2003) she bears an isolated plexiform neurofibroma shaped like an elongated sack fitting with the diagnosis of mosaic NF1.

#### 3.1.4 Federico da Montefeltro's facial lesions and dysplasia by Piero della Francesca [1472]

The Duke Federico III da Montefeltro [1422–1480 AD] was one of the most famous Italian Condottieri (military chieftains) of the Renaissance and Lord of Urbino. He was said to have lost his right eye during a tournament, carrying for the rest of his life a vast and disfiguring scar, so that it was necessary to portray him only on his “good” side: that is, the left profile (Roeck & Tonnesmann, 2009; Stevenson, 2021; Tommasoli, 1978; http://www.gallerianazionalemarche.it/palazzo-ducale/storia-della-famiglia-montefeltro/).

**FIGURE 4:**
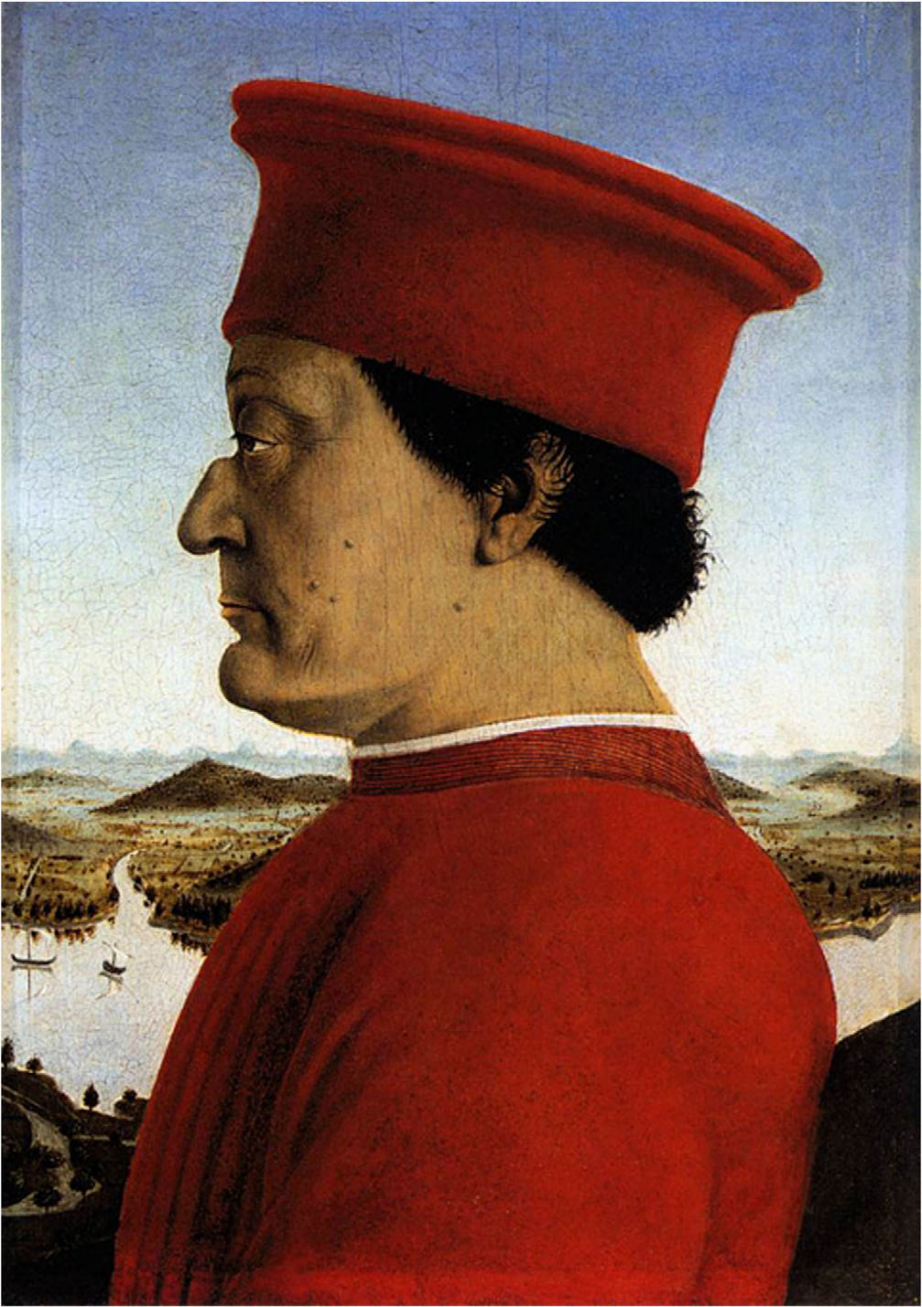
Portrait of the Duke Federico da Montefeltro by Piero della Francesca: note the multiple warts on his left face interpreted either as fibrous nodules or neurofibromas. Copyright © Galleria degli Uffizi, Firenze, Italy

Om and Om (2018) hypothesize, based on the portrait by Piero della Francesca (Figure 4) (https://www.uffizi.it/opere/i-duchi-di-urbino-federico-da-montefeltro-e-battista-sforza), that Montefeltro had several benign intradermal nevi, in the form of dome‐shaped, skin‐colored papules. These lesions, however, could also be attributed to small dermal neurofibromas, and the fact that he lost his right eye in a tournament has also been questioned, as he could have undergone surgical removal of an optic glioma and associated plexiform neurofibroma and dysplasia of the orbits. Notably, disruption of the orbit, either by trauma or surgery, may have invaded the root of the nose, whose shape is quite unusual (Ruggieri, Praticò, Caltabiano, & Polizzi, 2018).

#### 3.1.5 The infant of Chieri in Ambroise Paré's Des monstres et prodiges [1578 AD]

Ambroise Paré [1510–1590] was a famous French barber surgeon who cared for soldiers in battle. He was the official royal surgeon for several kings and is considered one of the fathers of surgery who developed surgical techniques and battlefield medicine, especially the treatment of wounds. He was also an anatomist and the inventor of several surgical instruments and authored many books on anatomy, surgery and medicine (Céard, 1971; Hambry, 1967).

**FIGURE 5:**
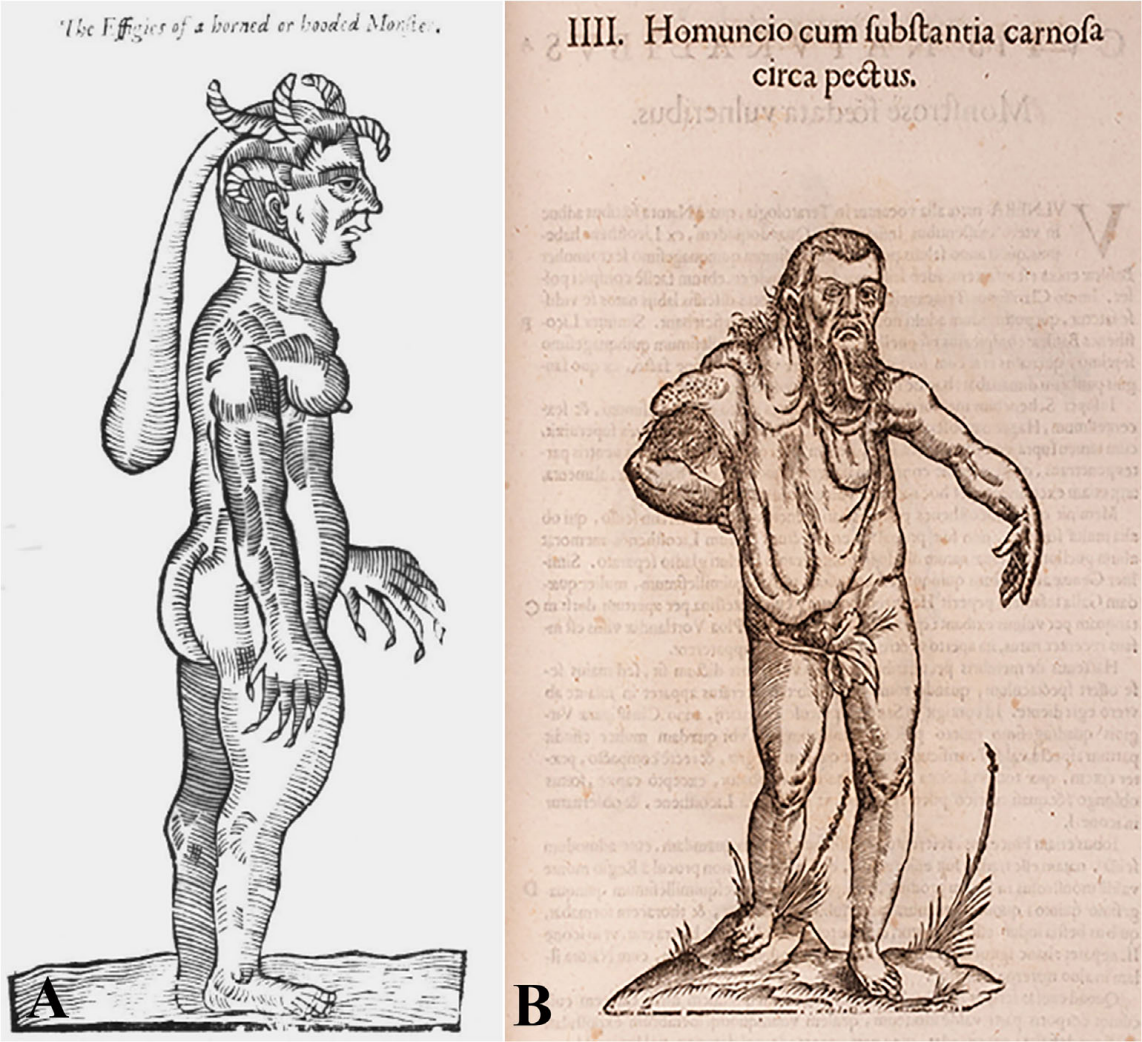
(a) The imaginative “infant of Chieri” by Ambroise Paré: the fleshy mass hanging down along the back was interpreted as a plexiform neurofibroma. Copyright © Wellcome Library London, UK; (b) Aldrovandi's “homuncio,” with enormous, flabby masses of flesh, hanging from his right side, likely representing a plexiform neurofibroma in mosaic neurofibromatosis type 1 (NF1). Copyright © Biblioteca Universitaria di Bologna, Italy

Among the various figures in his book *Des monstres et prodiges* (On Monsters and Marvels) (Pallister, 1995) is an infant born near Turin, Italy (Figure 5a) (Zanca & Zanca, 1977, 1980). The face “…was well‐proportioned in every way, but there were five horn‐like growths on the head and a long, fleshy mass hanging down from the head along the back “like a woman's hat…another double fleshy mass like a shirt collar was visible around the neck”. This fleshy mass hanging down along the back was diagnosed as a solitary plexiform neurofibroma (i.e., mosaic NF1) (Ruggieri & Polizzi, 2003, Ruggieri & Praticò, 2015). However, this severe anomaly is likely exaggerated and certainly inaccurate. Paré did not observe the case first‐hand, but obtained this drawing from a 1,578 pamphlet in Chambery (Pallister, 1995). Notably, Paré's sketch of this infant, with the less than believable claw‐like digits and horns, represents a fairly standard view of the relationship between human disorders and the conception of “monsters” during the 16th century (Brosius, 2010; Céard, 1971; Ruggieri & Polizzi, 2003; Ruggieri, Praticò, Caltabiano, & Polizzi, 2018).

#### 3.1.6 A large fleshy mass in Aldrovandi “homuncio” [Monstrorum Historia—1592]

Ulisse Aldrovandi [1522–1605], native of Bologna, was an Italian physician, philosopher, and naturalist (Caprotti, 1980;Simili, 2001; Tega, 2002). He was the driving force behind the construction of Bologna's botanical garden, one of the first in Europe, and of a most spectacular cabinet of curiosities, illuminating natural history and comprising some 18,000 “different examples of natural things,” and 7,000 “specimens of dried plants,” representing the “diversità di cose naturali” (“diversity of natural things”) (Simili, 2001; Tega, 2002, Wittkower, 1942).

In 1592, Aldrovandi recorded the extraordinary case of a man of low stature (homuncio), of Indian origin who presented enormous, flabby masses of flesh hanging from the left side of his head and trunk (Figure 5b) published posthumously (Aldrovandi, 1642). Some Italian Authors (Zanca, 1975; Zanca and Zanca, 1977, 1980; Zanca & Tagliavini, 1980) inferred that this misshapen man may represent a case of NF1, based on: (a) the appearance of the masses, which look like plexiform neurofibromas covered with hair, similar to the hairy nevi seen over plexiform neurofibromas in NF1; and (b) the short stature of the subject, a minor NF1 feature (Morse, 1999; Mulvhill, 1988). Madigan and Masello (1989) added a third NF1 feature, that is, macrodactyly of the left second toe and leg asymmetry secondary to scoliosis. Ruggieri and Polizzi (2003) proposed that the diagnosis of the homuncio would fit better with mosaic NF1 (Ruggieri & Praticò, 2015) rather than classical NF1 as the large fleshy mass (i.e., the plexiform neurofibroma) is solitary and there are no other NF1 stigmata either in the print or in the accompanying Latin text (Aldrovandi, 1642).

#### 3.1.7 The raised skin macules in Buffon's *Histoire Naturelle* girl [1749]

Comte Georges Louis Leclerc de Buffon [1707–1788] was a French naturalist, mathematician, cosmologist and encyclopedic author. Ten years after becoming the curator of the King's garden in Paris, he began publishing his 38 volumes of *Histoire Naturelle* (Natural History), a compendium of the natural history of animals and minerals, published over 40 years and continued posthumously (Hecht, 1989; Leclerc de Buffon, 2014; Madigan & Masello, 1989; Roule, 1924).

**FIGURE 6:**
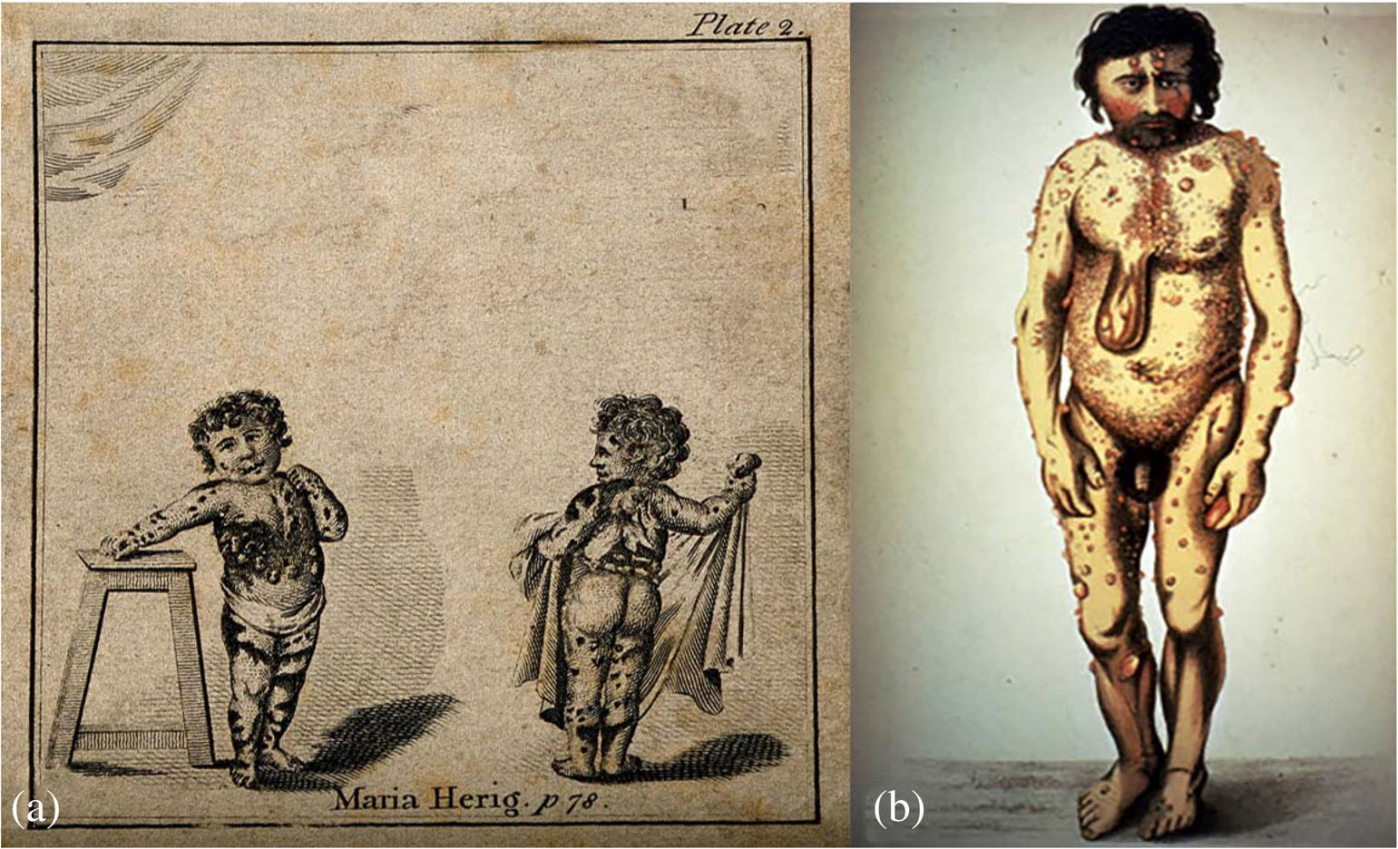
(a) Lithograph depicting Maria Herig with multiple dark leaf‐shaped areas of hyper pigmentation of the skin (likely café‐au‐lait spots) in the limbs and in the trunk and a large, raised “pigskin” encircling lesion over her trunk (likely a cutaneous plexiform neurofibroma or an epidermal nevus). Copyright © Wellcome Library London, UK; (b) von Tilenau's “Wart Man”.© Authors' personal collection

In one of the tables in the 1707–1788 edition there is a color drawing by B. de Bakker of a girl [Maria Herig] (Figure 6a, bottom)who shows different types of cutaneous anomalies: (a) multiple dark leaf‐shaped areas of hyperpigmentation in the limbs and trunk, possibly café‐au‐lait spots; (b) a large, “life vest‐” shaped lesion encircling the trunk, a possibly a diffuse cutaneous plexiform neurofibroma (Huson, 1994; Ruggieri & Polizzi, 2003). An alternative diagnosis is an epidermal sebaceous nevus with satellite lesions (Ruggieri, Praticò, Caltabiano, & Polizzi, 2018).

#### 3.1.8 The “Wart Man” of Tilesius von Tilenau [1793]

Tilesius von Tilenau (1769–1857) was a German physician, naturalist and explorer. In 1793, he reported on a case called the “Wart Man” (Tilesius von Tilenau, 1793). This patient, Johan Gottfried Reinhardt, was reported under the title “Case History of Extraordinary Unsightly Skin” and described as having “countless growths [fibrous tumours] on the skin, café‐au‐lait spots, macrocephaly, and scoliosis.” In a color reproduction (Figure 6b) of the original illustration accompanying the case presentation one can note a fleshy mass hanging forward toward the abdomen. The latter might be a pedunculated diffuse cutaneous plexiform neurofibroma (Ruggieri, Praticò, Caltabiano, & Polizzi, 2018).

## 4 TUBEROUS SCLEROSIS COMPLEX

Tuberous sclerosis (Bourneville, 1880–1881) is a complex multi‐system tumor‐predisposing syndrome characterized by the appearance of cutaneous hypomelanotic birthmarks and fibrous and capillary benign tumors of the skin and skin appendages including forehead plaques, facial angiofibromas, shagreen plaques, fibromas and ungual fibromas; cardiac rhabdomyomas, renal and/or systemic angiomyolipomas, retinal hamartomas; and brain malformations including cortical tubers, hamartomas, subependymal nodules, or subependymal giant cell astrocytomas (Curatolo, Bombardieri, & Jozwiak, 2008; Northrup et al., 2013). Affected people may develop seizures, mental and behavioral disabilities, and organ damage secondary to tumor progression (Krueger et al., 2013; Northrup et al., 2013; Ruggieri, Polizzi, et al., 2020).

### 4.1 The facial “végétations vasculaires” and “adenoma sebaceum”

**FIGURE 7:**
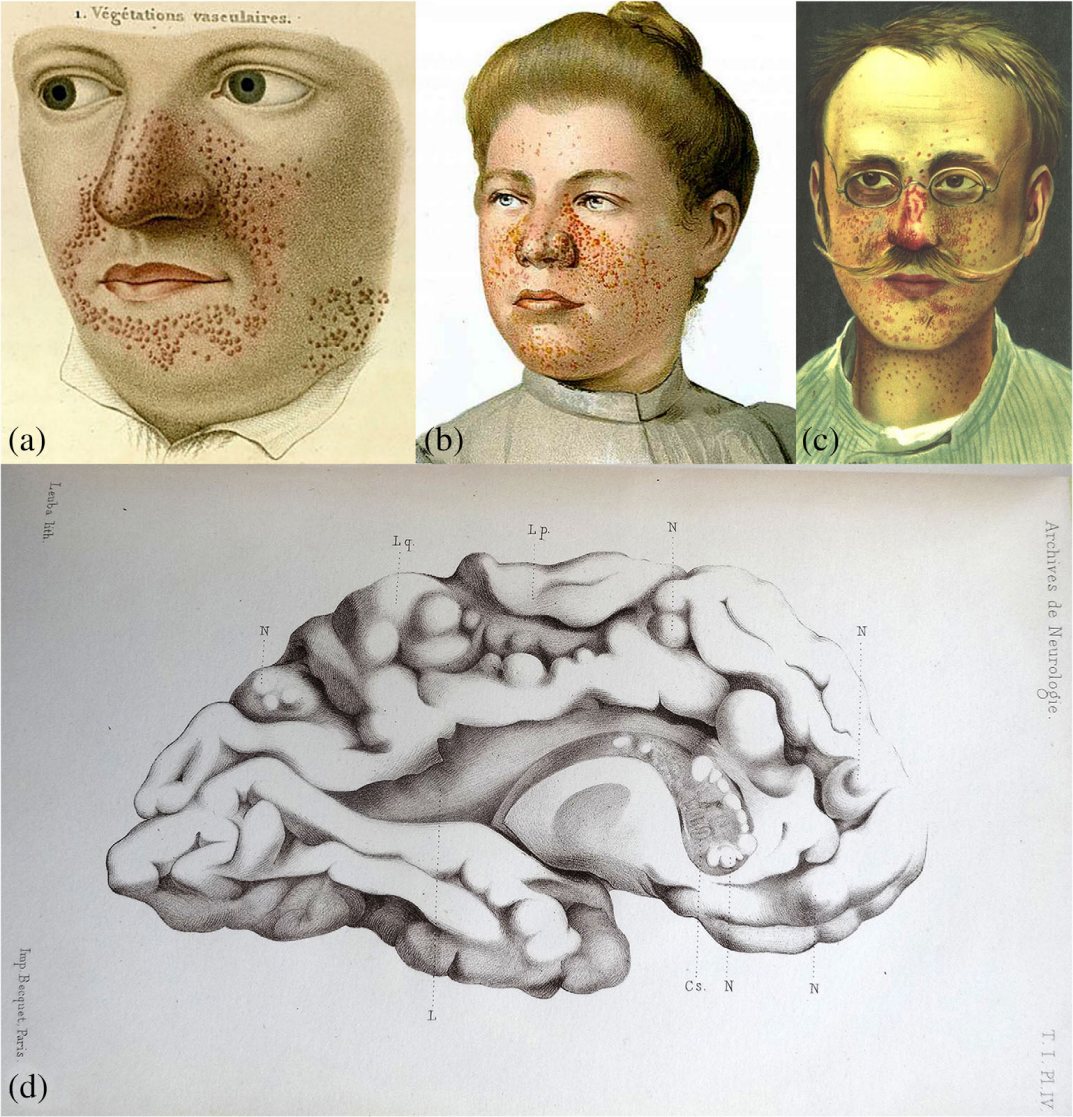
(a) A young man's face dotted with clusters of small, erythematous papules [végétations vasculaires] interpreted by Rayer as typical facial angiofibromas: Copyright © Wellcome Library, London, UK; (b,c) two 20th century prints depicting facial “adenoma sebaceum” (i.e., the old term used for the facial angiofibromas): Copyright © Wellcome Library, London, UK; (d) Drawings of the brain of the 15‐year‐old Marie: note the “sclerotic” cortical “tubers” and the “subependymal tumors” (nodules) of the lateral walls of the ventricles: Copyright © Wellcome Library, London, UK

An early possible illustration of one of the skin manifestations of tuberous sclerosis, facial angiofibromas, is given in 1835 by the French physician, pathologist, physiologist, parasitologist and dermatologist Pierre François Olive Rayer [1793–1867] (Théodoridès, 1995) in his atlas of skin disorders entitled *Traité Teorique e Pratique des Maladies de la Peau* (Theoretical and Practical Treatise on Skin Diseases) (Rayer, 1835) (https://www.cppdigitallibrary.org/collections/browse). On page 20, Figure 1 (Figure 7a) shows a young man's face dotted with clusters of small, erythematous papules [végétations vasculaires] with a characteristic distribution and similar appearance to the typical facial angiofibromas: “…small… of vascular appearance, widespread growths distributed on the nose and around the mouth….” No mention was made of any medical condition associated with the skin disorder (Curatolo, 2003; Jay, 1999).

Drawings and portraits of women (Figure 7b) and men (Figure 7c) represented with facial angiofibromas [at that time known as “adenoma sebaceum” as it was believed that the sebaceous glands were the source of the problem] (Balzer & Ménétrier, 1885; Curatolo, 2003; Gomez, 1995; Hallopeau & Leredde, 1885; Jay, 1999; Pringle, 1890) were common at the turn of the 19th century.

### 4.2 “Sclerotic islands and tumors” in the lithographs by Eduard Brissaud [1880]

The French physician, neurologist, neuropathologist and later Paris councilman and Deputy member of the French Parliament, Desiré Magloire Bourneville [1833–1910] provided the first (Bourneville, 1880–1881) and second (Bourneville & Brissaud, 1881) detailed clinical and neuropathological description of tuberous sclerosis in two children, Marie and George. The 15‐year‐old Marie, hospitalized in Paris at La Pitié Salpetrière since the age of 3 years and who had died secondary to the complications of status epilepticus, had the typical skin lesions of tuberous sclerosis and a history of severe psychomotor delay, learning and language difficulties, behavioral stereotypies, spastic hemiplegia and recurrent episodes of status epilepticus since the age of 2 years and was eventually declared a” hopeless case.” At postmortem examination, her brain revealed various whitish, opaque, dense, hypertrophic sclerotic islands (i.e., cortical tubers) alongside small sclerous clusters of tumors in the external walls of the lateral ventricles (i.e., subependymal nodules) (Brigo et al., 2018; Gomez, 1995). To describe the peculiarity of this cerebral pathology Bourneville coined the term “Sclérose tubéreuse des circonvolutions cérébrales” (tuberous sclerosis of the cerebral circonvolutions) and asked his friend and colleague Eduard Brissaud [1852–1909] to depict Marie's brain findings (Figure 7d).

## 5 HYPOMELANOSIS OF ITO AND THE LINES OF BLASCHKO

There is a heterogeneous group of disorders, so far attributed to chromosomal abnormalities, which are characterized by the occurrence of hypopigmented and hyperpigmented skin birthmarks in the form of whorls, streaks, V‐shaped, or S‐shaped alterations following the so‐called Lines of Blaschko, which represent a nonrandom, nonvisible embryological system of lines followed by pigmentary cells during development (Blaschko, 1901; Bolognia, Orlow, & Glick, 1994; Happle, 1993, 2014; Kromann, Ousager, Ali, Aydemir, & Bygum, 2018; Ruggieri, Polizzi, et al., 2020; Salas‐Labadía et al., 2019). These disorders have been collectively named “pigmentary mosaicism”; however, some individuals with hypopigmented abnormalities along the lines of Blaschko, may also have additional associated systemic involvement affecting principally the eyes, the musculoskeletal and nervous systems, thus configuring a complex neurocutaneous syndrome known as hypomelanosis of Ito (Ito, 1952), whose manifestations are cataracts, scoliosis, seizures, cognitive deficits, speech/language deficits, hypotonia, or abnormal EEGs (Ruggieri & Pavone, 2000; Ruggieri, Polizzi, et al., 2020).

### 5.1 Alfred Blaschko and his system of lines: A precursor of “mosaicism”

**FIGURE 8:**
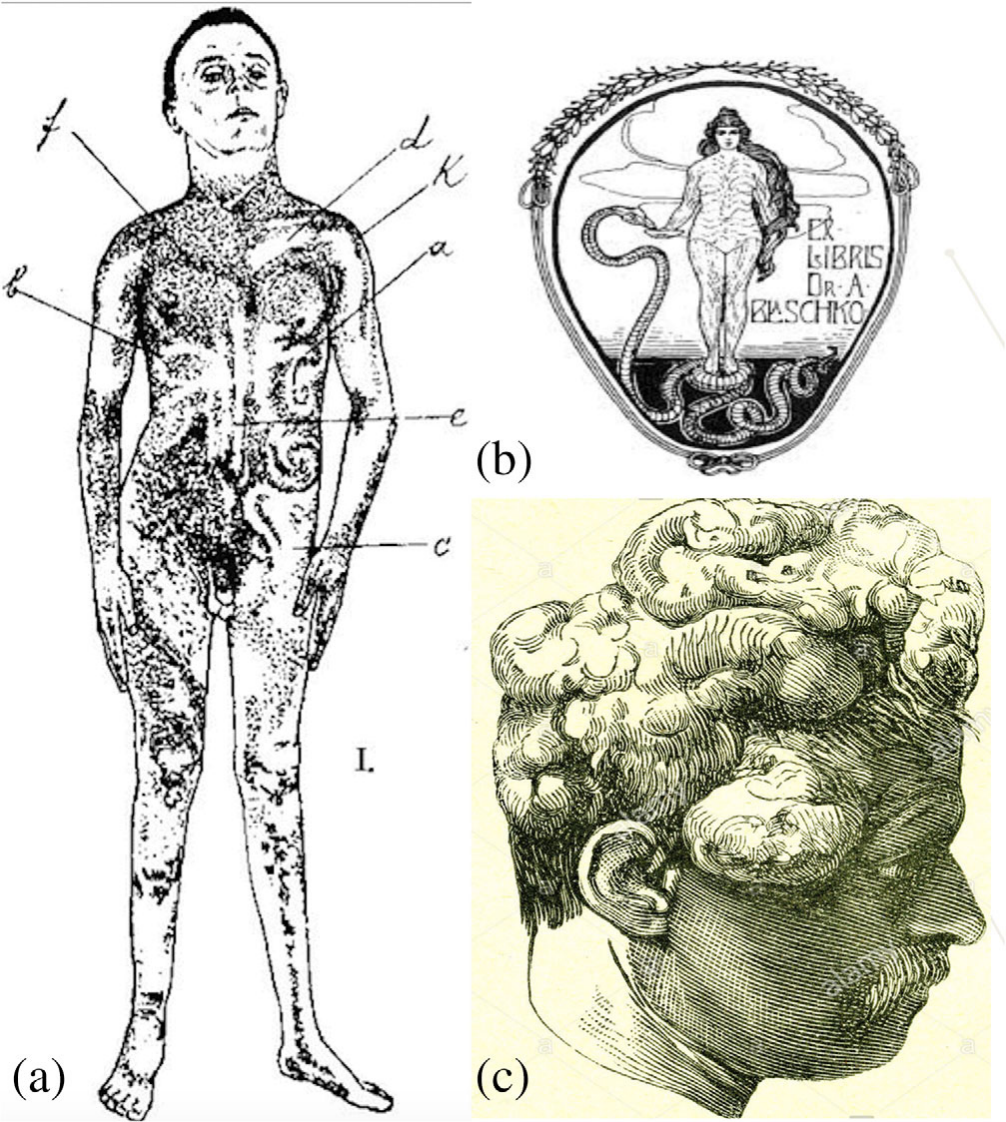
(a) One of the original drawings by Alfred Blaschko, depicting a child with the typical epidermal nevus lesions arranged according to specific lines of distribution of pigment, since called after his name and (b) Alfred Blaschko's bookplate, showing Hygieia, the Greek goddess of health, with the Blaschko lines superimposed. Copyright © The Cushing‐Whitney Medical Library, Yale University; (c) a print from a bookTreatise of Medicine [1889], depicting a man with multiple lipomas likely representing encephalocraniocutaneous lipomatosis

Alfred Blaschko [1858–1922] was a private practitioner of dermatology in Berlin whose interests ranged from leprosy to occupational skin diseases (Bolognia et al., 1994). In 1901, he presented his findings on the distribution patterns of linear skin disorders at the German Dermatological Society Meeting, in Breslau (Blaschko, 1901; https://archive.org/stream/dienervenverteil00blas#page/n5/mode/2up). He examined more than 140 patients with linear lesions such as epidermal nevi, sebaceous nevi, and nevus lipomatosus and carefully transposed the pattern in each patient onto dolls and statues used for illustrating his 1901 book (Figure 8a) (Blaschko, 1901). A composite diagram of these distribution patterns was then drawn that has subsequently been referred to as the Lines of Blaschko and applied in the liberty style cover of Blaschko's posthumous book (Figure 8b) (Happle, 1993).

## 6 ENCEPHALOCRANIOCUTANEOUS LIPOMATOSIS [HABERLAND SYNDROME]

This mosaic neurocutaneous syndrome with lipomatous changes (Haberland & Perou, 1970) is characterized by the occurrence of multiple lipomas in the zygomatic/fronto‐temporal area, nevus psiloparus, alopecic fatty tissue, nonscarring alopecia, focal dermal hypoplasia/aplasia, periocular skin tags, and hyperpigmentation along Blaschko's lines Additional features include epi‐bulbar choristoma, bone cysts, jaw tumors, extensive intracranial/intraspinal lipomas, cerebral asymmetry, arachnoid cysts, enlarged ventricles, leptomeningeal angiomatosis/capillary malformation, low‐grade gliomas, seizures, and intellectual disability (Levy & Massey, 2015; Ruggieri, Polizzi, et al., 2020).

### 6.1 Cranial protuberances in a 20th century medical treatise [1910]

A lithograph from a 20th century bookTreatise of Medicine shows an illustration (Figure 8c) of the head and face of a man with large, rounded masses protruding from his head, supposedly consisting of multiple lipomas within the context of encephalocraniocutaneous lipomatosis.

## 7 CONCLUSION

Neurocutaneous syndromes have been observed in numerous types of artworks through the millennia. We hope that readers will appreciate the art itself and the way of depicting and looking at these medical entities across the centuries and, ultimately, to view the humanity of the persons portrayed rather than their disorder, seen in the past as a “medical curiosity.”

## CONFLICT OF INTEREST

None.

## Data Availability

Data are available upon request to the author.
